# *In planta *transient expression as a system for genetic and biochemical analyses of chlorophyll biosynthesis

**DOI:** 10.1186/1746-4811-2-15

**Published:** 2006-09-05

**Authors:** Ruairidh JH Sawers, Phyllis R Farmer, Peter Moffett, Thomas P Brutnell

**Affiliations:** 1 Laboratory of Plant Genetics, University of Geneva, Sciences III, 30 Quai Ernest-Ansermet, 1211 Geneva 4, Switzerland; 2 Boyce Thompson Institute, Cornell University, Tower Road, Ithaca, NY 14853, USA

## Abstract

**Background:**

Mg chelatase is a multi-subunit enzyme that catalyses the first committed step of chlorophyll biosynthesis. Studies in higher plants and algae indicate that the Mg chelatase reaction product, Mg-protoporphyrin IX plays an essential role in nuclear-plastid interactions. A number of Mg chelatase mutants have been isolated from higher plants, including semi-dominant alleles of *ChlI*, the gene encoding the I subunit of the enzyme. To investigate the function of higher plant CHLI, bacterial orthologues have been engineered to carry analogous amino acid substitutions to the higher plant mutations and the phenotypes examined through *in vitro *characterization of heterologously produced proteins. Here, we demonstrate the utility of a transient expression system in *Nicotiana benthamiana *for rapidly assaying mutant variants of the maize CHLI protein *in vivo*.

**Results:**

Transient expression of mutant maize *ChlI *alleles in *N. benthamiana *resulted in the formation of chlorotic lesions within 4 d of inoculation. Immunoblot analyses confirmed the accumulation of maize CHLI protein suggesting that the chlorosis observed resulted from an interaction between maize CHLI and endogenous components of the *N. benthamiana *chlorophyll biosynthetic pathway. On the basis of this assay, PCR-based cloning techniques were used to rapidly recombine polymorphisms present in the alleles studied allowing confirmation of causative lesions. A PCR-based mutagenesis was conducted and clones assayed by transient expression. A number of novel allelic variants of maize Zm*ChlI *were generated and analyzed using this assay, demonstrating the utility of this technique for fine mapping.

**Conclusion:**

Transient expression provides a convenient, high-throughput, qualitative assay for functional variation in the CHLI protein. Furthermore, we suggest that the approach used here would be applicable to the analysis of other plastid-localized proteins where gain-of-function mutations will result in readily observable mutant phenotypes.

## Background

Transient expression systems provide a rapid and flexible platform for the functional analysis of proteins in native, or near-native, environments [1]. In addition, expression can be localized to specific tissues at specific developmental stages. The use of *Agrobacterium tumefaciens *to transfer recombinant plant and viral sequences into plant leaves is now routine and has been applied to transgenic complementation, promoter analysis and protein production [2-7]. A liquid culture of the recombinant bacterial strain is infiltrated into the leaves of a host plant (typically *Phaselous vulgaris *or a member of the genus *Nicotiana*). Following infiltration, recombinant T-DNA molecules are transferred to the nucleus of host plant cells. Stable transformation requires integration of T-DNA sequences into the plant genome. However, transient expression may also result from non-integrated T-DNA copies that persist in the nuclei of transfected cells [4]. Transgene expression and protein accumulation is localized to the site of infiltration and usually peaks 40–72 h post-infiltration. A strikingly successful application of *in planta *transient expression has been the characterization of signaling events involved in the initiation of a type of programmed cell death termed the hypersensitive response that is associated with pathogen resistance (e.g. [8]). The easily scored necrotic phenotype associated with the hypersensitive response has facilitated the analysis of protein function among natural occurring allelic variants and both targeted and randomly generated mutants (e.g. [9,10]). In this study, we demonstrate the application of these techniques to the study of chlorophyll biosynthesis. We demonstrate the utility of agroinfiltration in the dissection of Mg chelatase I function and suggest that this technique will have broad application in dissecting the function of many plastid-localized proteins.

The first committed step of chlorophyll synthesis is catalysed by magnesium chelatase (Mg chelatase), a hetero-oligomeric enzyme that inserts a magnesium ion into the cyclic tetrapyrrole protoporphyrin IX [11,12]. The enzyme consists of three subunits that are designated ChlI, ChlD and ChlH in higher plants [13,14]. We have recently characterized a number of semi-dominant alleles of the maize *Oil yellow1 *(*Oy1*) mutant and linked the mutant phenotype to variation in the Zm*ChlI *gene [15]. Homozygous *Oy1 *mutant seedlings are yellow in color and accumulate little or no chlorophyll, while heterozygous individuals exhibit a pale green phenotype intermediate to that of wild-type plants and homozygous mutants [16]. It has previously been suggested that the pale green phenotype of plants heterozygous for semi-dominant alleles of *ChlI *results from the formation of mixed oligomeric structures containing both mutant and wild-type CHLI subunits [15,17]. Thus, it was postulated that expression of mutant alleles in a wild-type background might induce chlorosis and provide an easily scored visual phenotype.

The use of transgenics to study gene function in maize is limited by existing transformation technology. The generation of stable transgenic lines is time consuming and costly and consequently is predominantly used to generate a small number of events, usually in the context of targeted agronomic manipulation. The use of a transient expression system is therefore particularly desirable. Although transient expression in leaf protoplasts [18] and microprojectile bombardment [19] have been successfully used in maize, they generate relatively small populations of transformed cells and are not necessarily applicable to the study of mature plant phenotypes. Therefore, we have used transient expression in *N. benthamiana *to study variation in maize Zm*ChlI*. We show that expression of mutant forms of the maize CHLI protein will induce localized chlorosis in mature leaves of *N. benthamiana *and demonstrate the utility of this approach in precisely defining the genotypic variation underlying dominant mutant phenotypes. Additionally, we transiently express variants of the *Synechocystis *ChlI protein in *N. benthamiana *and show a degree of functional conservation between bacterial and plant Mg chelatase components. Finally, we demonstrate the utility of transient expression in the screening of a population of mutagenized clones and the identification of novel allelic variants.

## Results and discussion

### Transient expression of mutant variants of maize CHLI in *Nicotiana benthamiana *results in localized chlorosis

**Figure 1:**
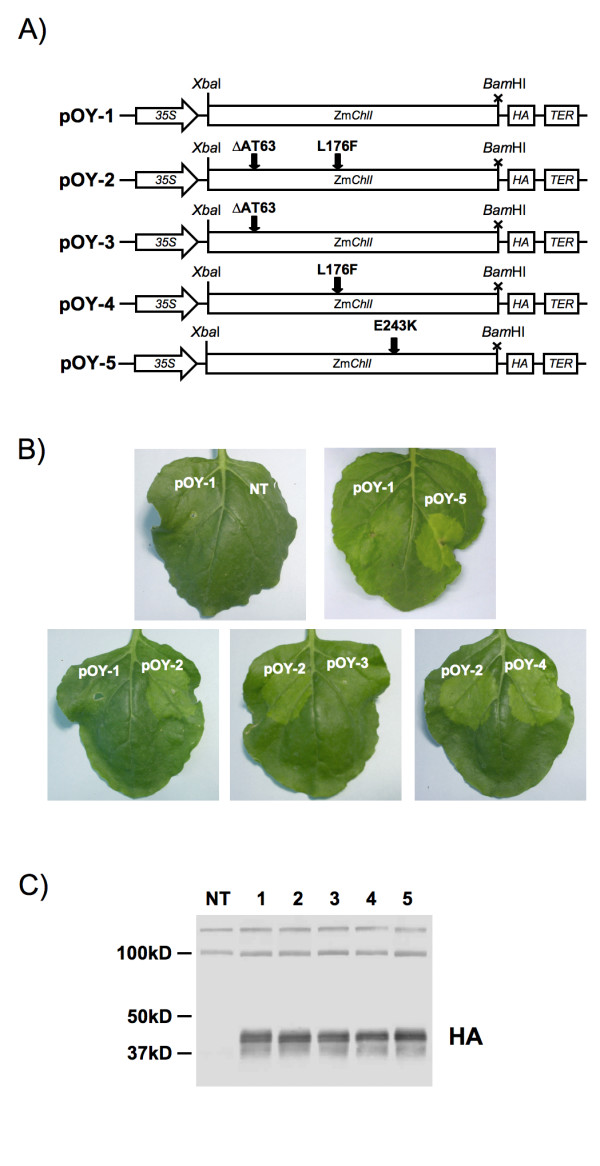
**Transient expression of semi-dominant Zm*ChlI *alleles in *N. benthamina***. A) Schematic of expression constructs, showing orientation of the 35S promoter (35S), maize chelatase I gene (Zm*ChlI*), HA-epitope tag (HA) and 35S terminator (TER) sequences. Wild-type Zm*ChlI *and a number of variants carrying polymorphisms present in semi-dominant mutant alleles were expressed in *N. benthamiana*. See text and Table 1 for details. B) Leaves of *N. benthamiana *12 days post-infiltration. Each leaf was inoculated with two constructs. C) Immunoblot analysis of ZmCHLI accumulation following infiltration with pOY-1 to 5 (lanes 1–5, respectively) or from non-transformed (NT) tissues. Immunoblot was challenged with anti-HA antibodies. The predicted size of the mature CHLI-HA fusion is approximately 41 kDa. A cross-hybridizing protein recognized by the HA antibody at approximately 100 kDa was used as a loading control.

Previous characterisations of two semi-dominant alleles of the maize *Oy1 *locus established that the chlorotic phenotype associated with *Oy1 *mutants was due to variation in the Zm*ChlI *gene [15]. In order to precisely define the polymorphisms within Zm*ChlI *that were causative of the disruption in chlorophyll biosynthesis, the Zm*ChlI *cDNAs were isolated from a wild-type maize line (W22 inbred) and two mutant lines (*Oy1-N700 *and *Oy1-N1989*) and expressed in *N. benthamiana*. Zm*ChlI *sequences were cloned into a derivative of the pBIN61 binary vector that contains a single 35S CaMV promoter sequence and provides a C-terminal HA-eptitope tag to the recombinant fusion protein (Figure 1; [9]). Constructs were then transformed into *Agrobacterium *and introduced into mature leaves using infiltration (see Methods). Leaf tissues were inspected at 4 to 9 days post-inoculation for the development of localized chlorosis around the point of infiltration.

An initial concern was that expression of the wild-type Zm*ChlI *might result in chlorosis, either via gene silencing of the endogenous *N. benthamiana ChlI *gene or through disruption of the proper stoichiometry of chlorophyll synthesis. However, no obvious phenotype was observed following expression of the wild-type maize sequence (Figure 1, pOY-1). Immunoblot analysis using an anti-HA antibody confirmed the accumulation of wild-type ZmCHLI following infiltration (Figure 1C). In contrast, expression of Zm*ChlI *coding sequences of the semi-dominant alleles *Oy1-N700 *(pOY-5) and *Oy1-N1989 *(pOY-2) induced chlorosis (Figure 1). These observations are consistent with *in vitro *biochemical data obtained for *Synechocystis ChlI *variants that were engineered to contain substitutions homologous to those present in the maize mutant alleles [15]. The ability of mutant maize CHLI variants to induce chlorosis in *N. benthamiana *suggests that the mechanism of action is conserved and that maize CHLI can interact with components of the *N. benthamiana *chlorophyll biosynthetic machinery. This observation is consistent with the high degree of conservation of CHLI proteins between plant species (mature maize and tobacco CHLI proteins are ~85% identical). Additionally, these observations demonstrate that the kinetics of transgene product accumulation and chlorophyll turnover are such that this system is appropriate for the study of chlorophyll biosynthesis and metabolism. Furthermore, these data identify the *Oy1 *alleles as both visual markers and as effectors of a localized and synchronous block in chlorophyll production. That is, within four days after infiltrations, we observed a block in chlorophyll accumulation across a field of leaf cells that are at a similar developmental stage.

### *In planta *expression allows rapid dissection of genetic variation

Transient expression of maize CHLI variants demonstrated that polymorphisms in the coding region of the maize Zm*ChlI *gene between mutant and wild-type (W22 maize inbred line) alleles were sufficient to induce chlorosis in *N. benthamiana*. The allele Zm*ChlI-Oy1-N700 *contains a single non-synonymous nucleotide change relative to wild-type sequence encoding the substitution of a glutamate residue at position 243 by lysine and therefore the results of transient expression are unambiguous. However, the allele Zm*ChlI-Oy1-N1989 *contains two polymorphisms relative to the W22 wild-type, corresponding to the deletion of residues 63 and 64 and the substitution of a leucine residue at position 176 by phenylalanine. In this instance, the presence of two linked polymorphisms in the same allele make it difficult to determine the functionally important lesion. Although maize mutant collections represent a valuable genetic resource, information is not always available concerning the background from which mutants were derived and therefore there may be no well-defined reference wild-type sequence. While multiple single nucleotide polymorphisms may be separated by genetic recombination, the frequency of intragenic recombination events often makes such an approach impractical [20]. However, using a transient expression assay, it is trivial to independently test the phenotypic effects of linked polymorphisms. Transient expression of variants containing one of two polymorphisms present in Zm*ChlI-Oy1-N1989 *(Figure 1, pOY-3 and pOY-4) demonstrated that the single L176F substitution was sufficient to induce chlorosis, while the two-residue deletion (ΔAT63) had no apparent effect. Interestingly, a dominant *ChlI *mutant in barley results from an analogous L to F substitution [17,21]. Furthermore, an equivalent L to F substitution in *Synechocystis *ChlI also results in a dominant mutant phenotype [15].

The two-residue deletion had not been previously tested using the *Synechocystis *system owing to poor conservation between maize and bacterial protein sequence in this region. In this instance, the utility of an *in planta *system to link genotypic and phenotypic variation is clear.

### Transient expression of a mutant Ss*chlI *allele demonstrates inter-specific conservation of enzyme function

**Figure 2:**
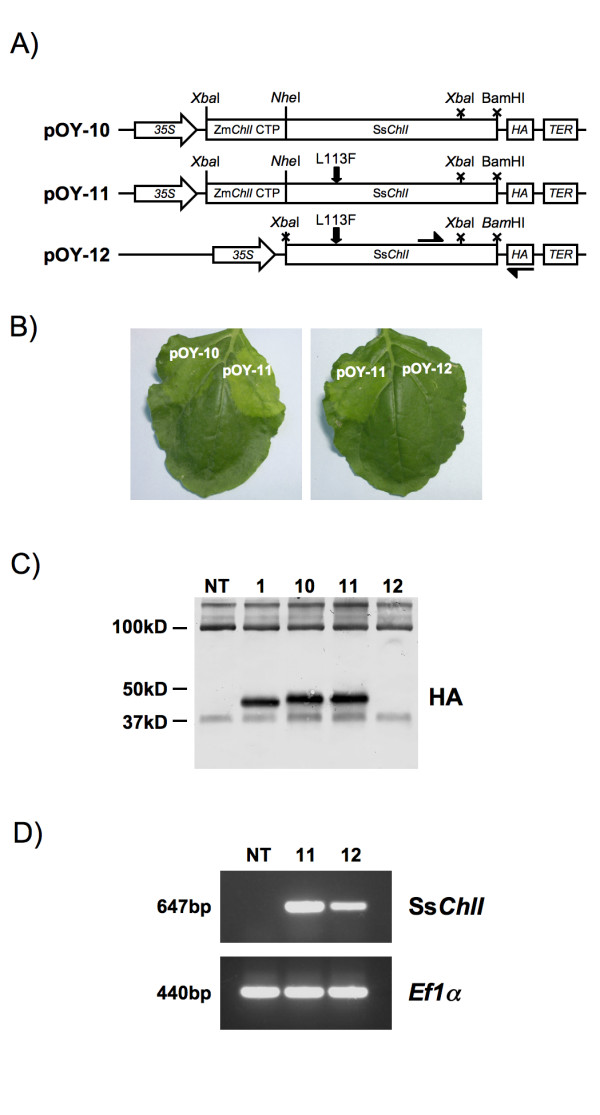
**Transient expression of Ss*ChlI *in *N. benthamiana***. A) Schematic of expression constructs (abbreviations as in Figure 1). Wild-type and mutant variants of Ss*ChlI *were expressed in *N. benthaminana *with the addition of an N-terminal CTP sequence. See text and Table 1 for details. The binding-sites of Ss*ChlI*-specific primers used in RT-PCR analysis are shown as arrows on the schematic of pOY-12. B) Leaves of *N. benthamina *12 days post-infiltration. No chlorosis was observed following infiltration with pOY-12. C) Immunoblot analysis of Ss*ChlI *protein accumulation following infiltration with pOY-1, pOY-10, pOY-11 and pOY-12 (lanes 1, 10, 11 and 12, respectively). A cross-hybridizing protein recognized by the HA antibody at approximately 100 kDa was used as a loading control. D) RT-PCR analysis of Ss*ChlI *transcripts in NT tissues or after infiltration with pOY-11 or pOY-12. Products amplified from an endogenous *Ef1α *transcript (440 bp) served as a control for cDNA synthesis.

Biochemical characterization of Mg chelatase function has largely been restricted to the *in vitro *kinetic analysis of bacterial proteins. Previous studies have sought to confirm and characterize higher-plant mutations by introducing homologous changes into bacterial orthologues [15,17]. In the case of *ChlI*, these studies have been possible due to the high degree of sequence similarity between plant and bacterial proteins [15]. However, in the absence of biochemical or structural data for higher plant CHLI, it is difficult to estimate the degree of mechanistic conservation between bacterial and plant enzymes. To investigate this further, *Synechocystis chlI *variants were transiently expressed in *N. benthamiana*. The N-terminal region of Zm*ChlI *predicted to encode the chloroplast tranist peptide (CTP) was fused to wild-type Ss*ChlI *(pOY-10) and to a Ss*ChlI *variant predicted to encode an amino acid substitution (Ss*ChlI-L113F*) homologous to the one observed in Zm*ChlI-Oy1-N1989 *(pOY-11). SsChlI-L113F has previously been shown in an *in vitro *assay to be non-functional in magnesium chelation and to competitively inhibit wild-type SsChlI function [15]. Transient expression of pOY-11 in *N. benthamiana *was found to condition chlorosis in a manner qualitatively similar to the homologous Zm*ChlI-Oy1-N1989 *variant (pOY-2) (Figure 2). This observation is consistent with mechanistic conservation between bacterial and higher-plant systems and suggests that both heterologous expression and homology modeling are valid approaches to the study of magnesium chelatase function.

Expression of Ss*ChlI-L113F*, lacking sequences encoding a CTP (pOY-12), did not result in chlorosis, suggesting that SsChlI-L113F disrupts chlorophyll synthesis by direct interaction with plastidic components, as is presumed to be the case for higher-plant dominant alleles [15,17]. However, immunoblot analysis revealed that levels of SsChlI protein accumulation were low in the absence of a CTP (Figure 2C). To confirm that DNA transfer had occurred and that the pOY-12 plasmid was functional, RT-PCR was used to assay the accumulation of Ss*ChlI *transcripts following infiltration with pOY-12 (Figure 2D). A product of 647 bp was amplified from cDNA prepared from pOY-12 infiltrated leaf tissue using a Ss*ChlI*-specific primer and a primer designed to the HA epitope sequence. A similar product was amplified from tissue infiltrated with pOY-11, but was not detected in non-transformed tissue. Amplification of *Ef1α *sequences served as a positive control for cDNA synthesis. These observations suggest that Ss*ChlI *transcripts are synthesized in pOY-12 transformed leaves but that SsChlI protein does not accumulate. It is interesting to note that, in this instance, the apparent stability and level of accumulation of a heterologous protein are dependent on translocation into the plastid and, potentially, on interaction with an endogenous protein complex in the stroma.

Somewhat surprisingly, expression of wild-type SsChlI with the N-terminal addition of the ZmCHLI CTP (pOY-10) resulted in mild chlorosis (Figure 2, pOY-10). Although, the effect was much stronger with the L113F mutation, it appears that the wild-type construct alone conditions a background level of chlorosis. In contrast, over-expression of maize wild-type CHLI does not result in chlorosis in this assay (pOY-1), despite its presumed incorporation into native complexes. Although inhibition of chelation has been observed following systemic, constitutive over-expression of wild-type ChlI [22], it appears unlikely that Ss*ChlI *would mimic this phenotype in a context where Zm*ChlI *would not. Potentially, divergence between bacterial and plant proteins allows interaction of heterologous components but results in compromised function of the resulting complex. In addition, immunoblot analysis showed that the major species of SsChlI is slightly larger than ZmCHLI (Figure 2C).

Mature ZmCHLI-HA is predicted to have a molecular mass of approximately 41 kD and full-length SsChlI-HA approximately 39.5 kD. The higher than expected observed mass of SsChlI may be due to a failure in processing the heterologous maize CTP following import of SsChlI variants. Non-processed SsChlI fusion proteins would be predicted to have a mass of approximately 45 kD. Failure to remove additional N-terminal residues may result in a partially functional protein that acts as a weak dominant negative. The ZmCHLI CTP was defined by computer-based prediction and by alignment with *Arabidopsis *protein sequence and has not been experimentally determined [15]. Importantly, the sequence used may not contain residues C-terminal to the site of processing that are required for processing-site recognition. Thus, while we cannot eliminate the possibility that the SsChlI and tobacco ChlI proteins are not functionally divergent, it seems more likely that the subtle chlorosis observed with the wild-type ScChlI is due to the lack of N-terminal processing of the transit peptide. These observations highlight the potential for dominant ChlI variants as markers not just for plastid localization but also for assaying the correct processing functional integration into endogenous components of the plastid machinery.

### Transient expression provides a system for mutational analysis of *ChlI*

**Figure 3:**
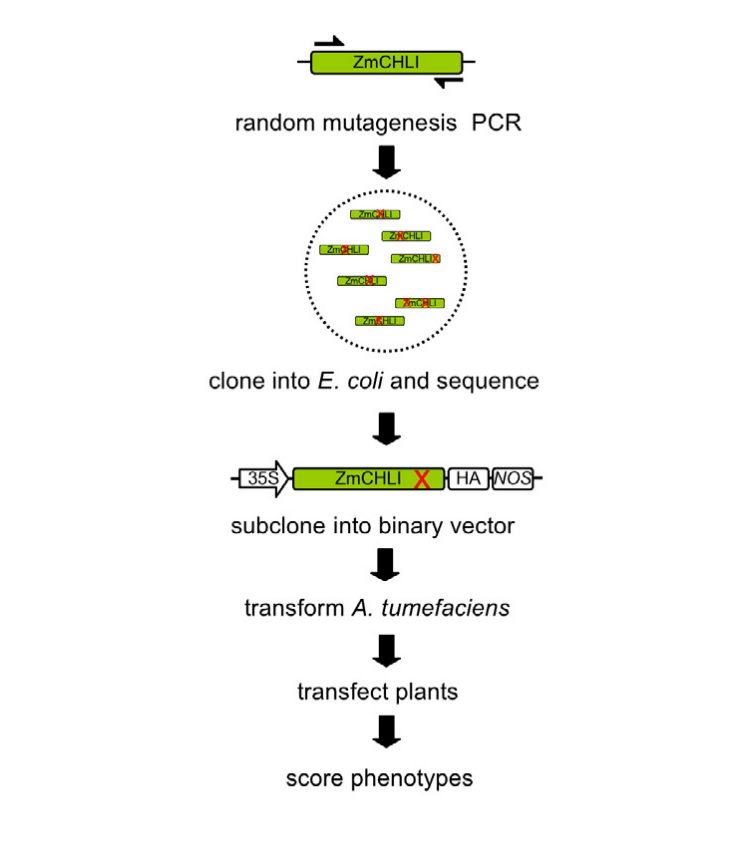
**PCR-based mutagenesis of Zm*ChlI***. Full-length Zm*ChlI *was amplified using a mutagenic PCR procedure and fragments cloned into the pGEM T-Easy. Plasmids were electroporated into *E. coli* and DNA from several clones sequenced. Fragments were then subcloned into the pBIN61 binary vector and transformed into *Agrobacterium*. Clones were grown overnight and expressed in *N. benthamiana *using agroinfiltration. Following infiltration, plants were screened for the development of chlorosis indicating the generation of novel dominant mutant variants.

**Figure 4:**
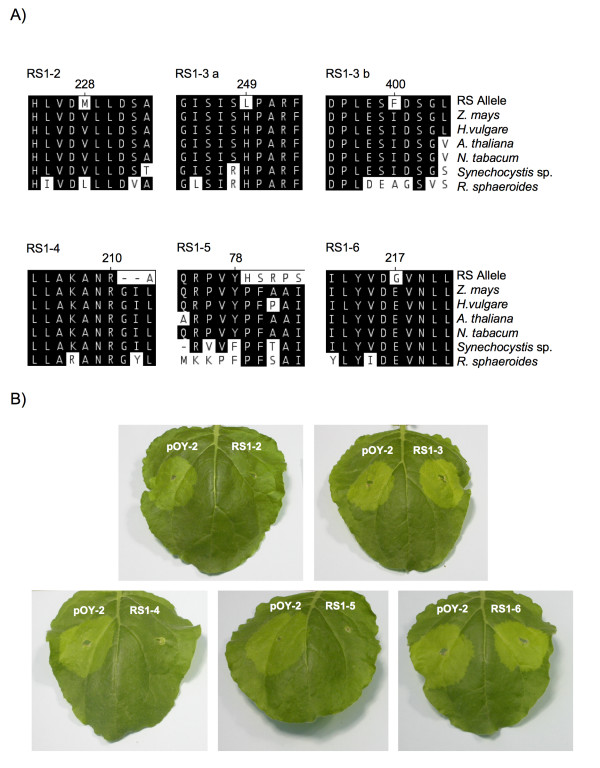
**Novel Zm*ChlI *alleles obtained from PCR-based mutagenesis**. A) Protein alignments showing lesions resulting from PCR-based mutagenesis. Recovered alleles compared to wild-type CHLI sequence from *Zea mays *(AAZ14052), *Hordeum vulgare *(DQ529207), *Arabidopsis thaliana *(NP_193583), *Nicotiana tabacum *(AAB97153), *Synechocystis *sp. (BAA17166), and *Rhodobacter sphaeroides *(ZP_00918218). Amino acid substitutions are denoted with the position of the change relative to the wild-type ZmCHLI sequence. B) Leaves of *N. benthamiana *12 days post-infiltration.

To identify novel allelic variation in maize ChlI, a PCR-based random mutagenesis procedure was used to introduce novel genetic variation (see Methods). Full-length Zm*ChlI *cDNA sequence was amplified by PCR under conditions that introduced random base-pair changes. Resulting populations were cloned into the vector pGEM T-Easy and a small number of clones sequenced. In a pilot study, five clones were selected that contained non-synonymous nucleotide changes, subcloned into the pBin61 vector and transformed into *Agrobacterium*. The clones were then infiltrated into *N. benthamiana *and leaves scored for chlorosis. A schematic of the procedure is shown in Figure 3. The predicted protein sequence of the five mutagenized clones is shown in Figure 4A. Vectors RS1–2 and RS1–6 contain single non-synonymous nucleotide changes, RS1–3 contains two non-synonymous changes and vectors RS1–4 and RS1–5 contain frame shift mutations.

As shown in Figure 4B, strong mutant phenotypes were observed in leaves of plants infiltrated with vectors RS1–3 and RS1–6. RS1–6 contains an E217G substitution within the highly conserved Walker B (DEAD box) domain [23] that likely disrupts the ATPase activity of the resulting protein. Significantly, the majority of previously characterized semi-dominant CHLI variants are deficient in ATPase activity [15]. RS1–3 contains both H249L and I400F substitutions. In this instance, it is not possible to unambiguously determine the causative lesion. However, PCR-based recombination, as described above for the Zm*ChlI-N1989*, would provide a method to obtain these two polymorphisms independently. A very subtle mutant phenotype was observed in plants infiltrated with the RS1–2 construct. RS1–2 carries a V228M substitution. Again the proximity to the Walker B domain suggests an effect on ATP binding or hydrolysis. No phenotypic effects were observed with the truncated forms RS1–4 or RS1–5. In all cases, a greater understanding of these alleles would be gained by introduction of the homologous substitutions into a bacterial ChlI and subsequent *in vitro *analysis. This is especially relevant to the analysis of the apparently weak RS1–2 mutant allele.

This pilot study highlights the applicability of transient expression to the identification of novel protein variants but also demonstrates the limitations with regard to assessing quantitative differences. We envisage that the greatest utility of this system would be as an initial genetic screen for phenotypic variation. The system described here also allows for secondary mutagenesis of known dominant alleles. We have previously converted a dominant mutant variant CHLI into a recessive form by targeted amino acid substitution (Ruairidh Sawers, Jo Viney and Neil Hunter, University of Sheffield, UK, unpublished results). It is anticipated that other such events could be recovered by random mutagenesis.

### General conclusions on the potential of this transient expression system for the study of chlorophyll biosynthesis

Although genetic studies have been instrumental in the identification chlorophyll biosynthetic genes, the analysis of chelatase function has largely been restricted to *in vitro *kinetic analysis of wild-type protein function. However, an extensive collection of photosynthetic mutants in barley and maize exist [15,17,21,24]. Of particular interest with regard to enzymatic function are a number of semi-dominant alleles of *ChlI*. The cloning of several of these alleles [15,17] and the subsequent introduction of observed variation into bacterial proteins for *in vitro *analysis has demonstrated the potential of mutant variants in extending understanding of these enzymes. The observation that transient expression of dominant *Oy1 *alleles is sufficient to induce chlorosis opens up the possibility for the identification of further alleles, not only of *ChlI *but also of other components of the pathway. As described here, this approach is most suitable for the identification of dominant and semi-dominant variants. Dominant variants are most likely to be isolated where there is some form of protein-protein interaction underlying the process. For this, the *ChlD *and *ChlH *subunits of Mg chelatase make attractive targets. Additionally, it has been suggested that GUN4 [25] and CHLM may also physically interact [26]. While such interactions may be transient and difficult to characterize *in vitro *using wild-type components, semi-dominant mutant forms may reveal, and even stabilize, complexed states.

## Conclusion

Transient expression of dominant mutant alleles of *ChlI *in *N. benthamiana *demonstrated that such a system is applicable to the study chlorophyll synthesis and turnover. Causative lesions were confirmed for two maize alleles Zm*ChlI-Oy1N700 *and Zm*ChlI-Oy1N1989*. In addition, three potential novel alleles of Zm*ChlI *were identified. Although *in vitro *kinetic analyses are required for quantitative characterization of enzymatic variants, the investment required makes such analyses most appropriate to the study of a small number of variants. In contrast, the transient expression system described here allows the rapid, qualitative analysis of a large number of variants. A combination of these two approaches offers the possibility for the large-scale isolation and characterization of allelic variation from plant and bacterial species.

## Methods

### Construction of pOY vectors

The pBIN61-pOY binary vectors contain a series of *ChlI *fusions and derivatives under the control of the CaMV 35S promoter. All constructs contain a C-terminal HA epitope fusion and 35S terminator sequence. Coding sequences of pOY vectors were assembled and sequenced in pGEM-T Easy (Promega, Madison, WI) prior to sub-cloning to the binary vector pBIN61-RxHA [27] to generate pBIN61-pOY vectors. pBIN61-RxHA was digested with *Xba*I and *Bam*HI to release the *Rx *coding fragment. The pOY coding sequence was then introduced as an *Xba*I/*Bgl*II fragment.

Coding sequences for vectors pOY-1, pOY-2 and pOY-5 were PCR amplified directly from cDNA prepared from maize wild-type (W22 inbred), *Oy1-N1989 *and *Oy1-N700 *plants, respectively. Amplification reactions were performed using the primer CHLIFLF (5'-GGCACGACAGGTTTCCGACT-3') and the reverse primer CHLIFLR (5'-TGAAAGTAACAGAACAAGAGGGTTCC-3'). The full-length products were ligated into pGEM-T Easy (Promega), and subsequently used to transform *E. coli*. Plasmid DNA prepared from bacterial cultures containing the constructs was then used as template in PCR amplification using primers CHLIXBAFWD (5'-CTCCTCTCTAGAATGGCTTCCACCTTCTC-3') and the reverse primer CHLIBGLREV (5'-GAACAGATCTGCTAAAGACTTCATAAAACTTCTC-3') to introduce 5' *Xba*I and a 3' *Bgl*II sites (underlined).

The coding sequences for vectors pOY-3 and pOY-4 were generated using a two-step amplification strategy to engineer constructs with single lesions. To create pOY3, primers CHLIXBAFWD and CHLI55ATSEPMUT (5'-ATGGTGATCTTGGCGGTGGT-3') were used to amplify a 522 bp region from pOY2 incorporating the ΔAT63 lesion. In a separate reaction CHLIBGLREV and CHLI55FSEPMUT (5'-GTACCCATTCGCGGCCATC-3') were used to amplify a 1061 bp fragment from pOY1. To generate pOY-4, primers CHLIXBAFWD and CHLI55ATSEPMUT were used to amplify a 522 bp region from pOY1. In a separate reaction CHLIBGLREV and CHLI55FSEPMUT were used to amplify a 106 bp fragment from pOY2 to incorporate the L176F lesion. Products were then gel purified using a Gel Extraction kit (Qiagen, Valencia, CA) and resuspended in 50 μl of EB buffer. Approximately 20 ng of each purified product was used as template in a second amplification reaction together with primers CHLIXBAFWD and CHLIBGLREV to amplify a full-length synthetic gene. Products were sub-cloned to pBIN61-RxHA as described above.

Vectors pOY-10 and pOY-11 were generated by fusion of the Zm*ChlI *CTP to *Synechocystis *sequence. The vectors were assembled in pGEM-T Easy prior to sub-cloning to pBIN61-RxHA as *Xba*I/*Bgl*ll fragments as described above. Initially, the Zm*ChlI *CTP was amplified from wild-type template using the primer CHLIXBAFWD and the reverse primer CHLIXBABGL 5'-GTAGACAGATCTCAGATGGCTAGCGACATTGCAGACGGTGAATC-3' containing *Nhe*I and *Bgl*II restriction sites (underlined) and the fragment cloned to pGEM-T Easy. Wild-type (pOY-10) and mutant (pOY-11) sequence was amplified from previously described vectors [15] using the primers CHLINHE 5'-CATGTAGCTAGCATGACTGCCACCCTTGCCGCCCCCAGC-3' and CHLIBGLII 5'-CATGTAAGATCTAGCTTCATCGACAACGCCAAAAAC-3' to introduce 5' *Nhe*I and 3' *Bgl*II sites (underlined) used in sub-cloning to the vector containing the signal sequence. Prior to amplification, the template vectors were modified to remove an endogenous *Xba*I site present in the *SsChlI *gene in order to allow for the use of the common strategy of sub-cloning to pBIN61-RxHA as described above. The *SsChlI *coding sequences (wild-type and mutant) were excised from pET-9a [15] and cloned to the pGEM-T Easy (Promega) backbone as *Nhe*I/*Eco*RI fragments. A portion of the coding sequence was amplified from the 5' end to the endogenous *Xba*I site using the primers CHLIENDO 5'-AACCGGGAAATCCACTACCATTAGAGCTTT-3' and CHLISPE 5'-CATGTAACTAGTGGATCTTTCCGTAAACGATG-3' to introduce a *Spe*I site (underlined) at the position of the *Xba*I site. These products were then cloned as a *Bam*HI/*Spe*I fragments back to the pGEM-T Easy (Promega) template digested with *Bam*HI/*Xba*I. The compatible ligation of *Spe*I and *Xba*I ends resulted in a synonymous base pair change and the ablation of the endogenous site. The vector pOY-12 was built by digestion of pOY-11 with *Nhe*I and *Bgl*II and sub-cloning to *XbaI*/*Bam*HI digested pBIN61-RxHA.

### Transient expression

The pOY constructs, derived from pBIN61-RxHA, were transformed into *Agrobacterium tumefaciens *strain C58C1 carrying the virulence helper plasmid pCH32 [28]. *Agrobacterium*-mediated transformation was performed essentially as described by Bendahmane *et al*. [27]. *N. benthamiana *seedlings were sown in flats and grown under greenhouse conditions (24°C – 30°C) for 21 days. They were then transplanted to six inch pots and grown for an additional 7 to 10 days. Infiltrations were conducted in the laboratory under fluorescent light at 24°C – 28°C. After a 24 hr recovery in the laboratory, plants were returned to the greenhouse with supplemental lighting provided by 400 W metal halide lamps (average temperatures in the greenhouse varied between 26°C and 30°C). We found phenotypes were highly reproducible. In over 20 independent infiltrations using pOY-4 conducted on multiple leaves on multiple plants, we always observed chlorosis. Some subtle variation in phenotype was observed that was dependent on the developmental age of the leaf and temperature in the greenhouse. The most severe phenotypes were observed when leaf 6 or 7 was infiltrated when seedlings were 3 weeks old. At the time of infiltration leaves were approximately 2.5 to 4.0 cm in width.

### RT-PCR

Total RNA was made from approximately 75 mg of tissue using the RNeasy Plant Mini Kit (Qiagen, Valencia, CA). DNAse treatment was performed on a column during RNA extraction using the RNase-Free DNase Set (Qiagen) according to the manufacturer's protocol. Subsequent synthesis of cDNA was performed using the SuperScript First-Strand Synthesis System for RT-PCR (Invitrogen, Carlsbad, CA) with 2 μg total RNA in a volume of 10 μl. l μl of cDNA was used as template with the primers SsCHLIF (5'-CGAAGTTAACCTGTTGGACGATCAC-3') and HAR (5'-CGGCATAATCAGGTACATCATAAGGGTAG-3') to amplify a product of 647 bp. EF1α was amplified using the primers EF1aFor (5'-GGTGGTTTTGAAGCTGGTATCTCC-3') and EF1aRev (5'-CCAGTAGGGCCAAAGGTCACA-3'). The products were visualized on a 0.8% agarose gel run at 70 V for approximately 3 hours.

### Immunoblot analysis

Protein was extracted by grinding 3 square centimeters of infiltrated leaf tissue in 500 μl of extraction buffer [100 mM Tris pH 7.5, 150 mM NaCl, 1 mM DTT, 0.1% TritonX 100] with Protease Inhibitor Cocktail (Sigma-Aldrich, St. Louis, MO). Samples were then centrifuged at 17,900 rcf at 4° for 10 min. The supernatant, containing the soluble protein fraction, was then mixed with 10× loading buffer Z [125 mM Tris pH 6.8, 12% SDS, 10% Glycerol, 0.001% Bromophenol Blue], boiled at 95° for 3–5 minutes and loaded onto a Criterion Tris-HCL 12.5% acrylamide gel (Bio-Rad, Hercules, CA). After separation, the samples were electroblotted to Protran BA83 0.2 μm nitrocellulose membrane (Whatman, Schleicher & Schuell, Keene NH). The blots were rinsed twice in 1× PBS pH 7.5 and gently rocked for at least 1 hour in blocking buffer [5% nonfat milk powder in PBST (0.05% Tween 20 in 1× PBS)]. A rabbit anti-HA antibody (Sigma-Aldrich) was diluted 1:10,000 in blocking buffer and used to challenge blots for at least 1 hour. A goat anti-rabbit secondary antibody conjugated to horseradish peroxidase (Bio-Rad) was then used to amplify the signal. Visualization was accomplished using the Opti-4CN Substrate kit (Bio-Rad).

### PCR-based random mutagenesis

PCR-based mutagenesis was performed using the Diversify PCR Random Mutagenesis kit (Clontech, Mountain View, CA) according to the manufacturer's protocol for Buffer Condition 2. Wild-type Zm*ChlI *plasmid was used as PCR template in conjunction with the primers CHLIXBAFWD and CHLIBGLREV (described above). These conditions were used to introduce mutations at a rate of 2.3 substitutions/1000 bp. The mutagenised products were inserted into pGEM-T Easy vector and a subset were sequenced, analyzed, and subcloned into the pBin-61 Rx HA expression vector. The resulting plasmids were used to transform *Agrobacterium *and transient expression was accomplished as described above.

## Competing interests

The author(s) declare that they have no competing interests.

## Authors' contributions

RS and PF constructed pOY vectors, conducted infiltration experiments and drafted the manuscript. PF conducted RT-PCR experiments and performed protein blot and sequence analysis. PM provided technical advice on the expression system and helped to draft the manuscript. TB coordinated the study and helped to draft the manuscript. All authors have read and approved the final manuscript.

**Table 1:** pOy vectors

Vector	Description	Residue alterations	Protein detected	Phenotype
pOY-1	*ZmChlI *wild-type	-	yes	none
pOY-2	*ZmChlI-Oy1-N1989*	ΔAT63 L176F	yes	chlorosis
pOY-3	*ZmChlI-Oy1-N1989 *derivative	ΔAT63	yes	none
pOY-4	*ZmChlI-Oy1-N1989 *derivative	L176F	yes	chlorosis
pOY-5	*ZmChlI-Oy1-N700*	E243K	yes	chlorosis
pOY-10	*SsChlI *wild-type, N-terminal CTP fusion	ZmCHLI(1–54)-SsCHLI	yes	chlorosis
pOY-11	*SsChlI-L113F*, N-terminal CTP fusion	ZmCHLI(1–54)-SsCHLI-L113F	yes	chlorosis
pOY-12	*SsChlI-L113F*, no CTP fusion	L113F	no	none
